# Prognostic role of pretreatment platelet to lymphocyte ratio in urologic cancer

**DOI:** 10.18632/oncotarget.20147

**Published:** 2017-08-10

**Authors:** Jianfeng Wang, Yang Liu, Naiwen Zhang, Xuejie Li, Peng Xin, Jianbin Bi, Chuize Kong

**Affiliations:** ^1^ Department of Urology, The First Hospital of China Medical University, Shenyang 110001, P.R. China

**Keywords:** platelet to lymphocyte ratio, urologic cancer, prognosis, meta-analysis

## Abstract

The prognostic value of platelet to lymphocyte ratio (PLR) in urologic cancer does not reach a consensus. Herein, we performed the meta-analysis to determine the prognostic role of PLR in patients with urologic cancer. A literature search was performed in the PubMed, Embase, and Web of Science databases. Hazard ratios (HRs) were extracted to estimate the association between PLR and prognosis. A total of 20 articles comprising 6079 patients were included in this study. The pooled results showed that a high PLR was significantly associated with worse prognosis of overall survival (OS) in urologic cancer [HR=1.65, 95% confidence interval (CI) =1.37-1.99, P<0.01]. The result also indicated that an elevated PLR was significantly associated with poor OS in renal cancer (HR=1.88, 95% CI=1.39-2.55, P<0.01). In addition, the significant association between poor OS and elevated PLR in renal cancer was consistent regardless of treatment, cut-off value, sample size and study quality. Our result also indicated that an elevated PLR predicted shorter OS (HR=1.78, 95% CI=1.38-2.30, P<0.01) and cancer-specific survival (HR=2.02, 95% CI=1.24-3.29, P<0.01) in prostate cancer. In conclusion, an elevated PLR was a predictive indicator of poor survival in renal cancer and prostate cancer.

## INTRODUCTION

Urologic cancer is one of the most common of cancers worldwide, with an estimated incidence of 146,650 new cases and 32,190 deaths in United States in 2017 [1]. Until now, TNM staging is the most commonly used method to predict the prognosis and guide treatment in cancer. However, urologic cancer patients with the same TNM stage may have different clinical prognosis [2]. Thus, this leaves a large space for the development of additional biomarkers to predict the clinical outcome.

Recently, more and more evidence have reported that the development and prognosis of cancer are affected not only by cancer characteristics but also by host systemic inflammatory response [3, 4]. In clinical work, the inflammatory response can be evaluated by lots of biomarkers such as neutrophil to lymphocyte ratio (NLR), platelet to lymphocyte ratio (PLR) and C-reactive protein, etc.[5]. Now, NLR have been reported to be a prognostic predictor of urologic tumors such as bladder cancer, renal cell cancer, upper tract urothelial cancer (UTUC) and prostate cancer [6–10]. On the other hand, a growing body of evidence reports that a high PLR was a poor prognostic indicator in various types of cancers including lung cancer, colorectal cancer, gastric cancer and breast cancer [11–14]. But the prognostic value of PLR in urologic cancer does not reach a consensus. To the best of our knowledge, until now there was no a pooled study to assess the prognostic significance of PLR in urologic cancer.

In this study, we searched the relevant articles and conducted a pooled study to explore the prognostic value of PLR in urologic cancer including renal cancer, UTUC, bladder cancer, prostate cancer and adrenal cancer.

## RESULTS

### Studies characteristics and overall effect

**Figure 1:**
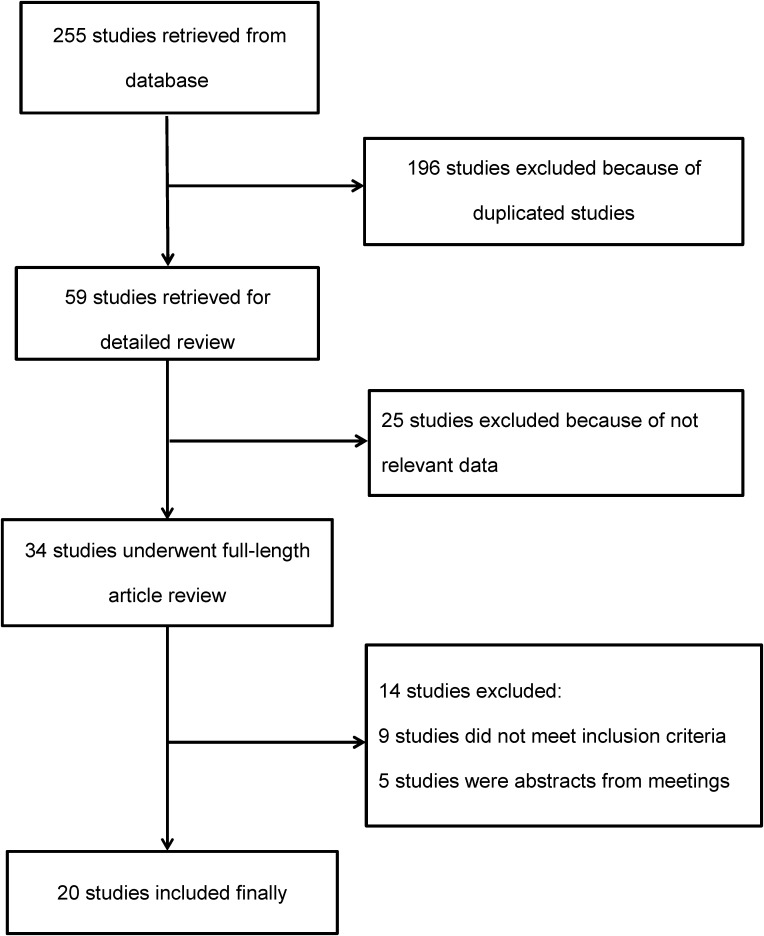
Flow diagram of article selection procedure

The literature search strategy yielded 255 potentially relevant studies and then 235 citations were excluded. Finally, a total of 20 articles were included in this study [15–34]. The flow diagram of study selection procedure is shown in Figure 1.

All these retrospective cohort studies were published in 2013 or later. Nine studies were performed in Asian populations, while 11 articles were based on Western population. Among these 20 studies, 8 studies focused on renal cancer, 4 for UTUC, 3 for bladder cancer, 4 for prostate cancer and 1 for adrenal cancer. The characteristics of these studies are shown in Table 1.

**Table 1:** Baseline characteristics of studies included in the meta-analysis

Author	Year	Country	No. of patients (M/F)	Age mean±SD/ median (range)	Cut off	Type of cancer	Duration of follow-upmean (range)	Surgery	Staging of TNM	Outcome	QS#
Dirican [15]	2013	Turkey	53(39/14)	61(40-79)	134	RC	34(5-142)	P	IV	OS	6
Fox [16]	2013	Australia	362(268/94)	62(19-84)	192	RC	NR	N	III/IV	OS	7
Keskin [17]	2014	Turkey	211(135/74)	61.18±11.81	151	RC	24	Y	I/II/III/IV	OS	6
Gunduz [18]	2015	Turkey	100(79/21)	58(33-95)	210	RC	32.7	N	IV	OS,PFS	6
Lucca [19]	2015	Austria	430(257/173)	65.5(57-73)*	145	RC	40(17-73)*	Y	I/II/III	DFS	7
Park [20]	2016	Korea	63(52/11)	63.1(56.0-70.5)*	150	RC	17.5(9.2-28.4)*	N	IV	OS,PFS	5
Chrom [21]	2017	Poland	321(215/106)	62(22-85)	157	RC	55.5	Y	IV	OS	6
Hu [22]	2017	China	484(278/206)	56(21-81)	185	RC	36	Y	I/II/III/IV	OS	8
Kim [23]	2015	Korea	277(218/59)	63.7(29.5-90.0)	150*	UTUC	NR	Y	I/II/III	DFS	5
Huang [24]	2016	China	481(311/170)	NR	241.2	UTUC	NR	Y	I/II/III	OS,CSS	5
Dalpiaz [25]	2016	USA	180(109/71)	70(62.7-77.2)*	150	UTUC	30	Y	I/II/III	OS,CSS	8
Song [26]	2016	China	140(86/54)	67(39-81)	128	UTUC	NR	Y	NR	DFS,PFS	7
Lee [27]	2015	UK	226(174/52)	75 (65-81)*	218	BC	NR	Y	I/II	OS	7
Zhang [28]	2016	China	124(100/24)	65(30-78)	140	BC	NR	Y	I/II/III/IV	OS	8
Kang [29]	2016	Korea	1551(1302/249)	65(57-72)*	124	BC	52(27-82)*	Y	0a/0is/I	OS,CSS	6
Langsenlehner [30]	2015	Austria	374(374/0)	68±7.1	190	PC	87	NR	NR	MFS,CSS,OS	6
Li [31]	2015	China	103(103/0)	66.1±6.9	150	PC	36	NR	NR	OS	5
Lolli [32]	2016	Italy	230(230/0)	74(45-90)	210	PC	29(1-55)	NR	IV	OS	7
Wang [33]	2016	China	290(29/0)	75(67-79)*	117.58	PC	37.0(24.0-50.3)*	NR	NR	PFS,CSS,OS	7
Bagante [34]	2015	USA	79(46/33)	NR	190	AC	NR	Y	I/II/III/IV	RFS,DSS	6

*The rage is inter-quartile range (IQR). #quality of study was judged based on the Newcastle-Ottawa Scale.

AC: adrenal cancer; BC: bladder cancer; CSS: cancer-specific survival; DFS: disease free survival; DSS: disease-specific survival; N: none of patients accept the surgery; NR: not reported; MFS: metastases–free survival; OS: overall survival; RFS: recurrence-free survival; P: part of patients accept the surgery; PC: prostate cancer; PFS: progression-free survival; RC: renal cancer; SD: standard deviation; QS: quality of study; UTUC: upper tract urothelial carcinoma; Y: all of patients accept the surgery.

**Figure 2:**
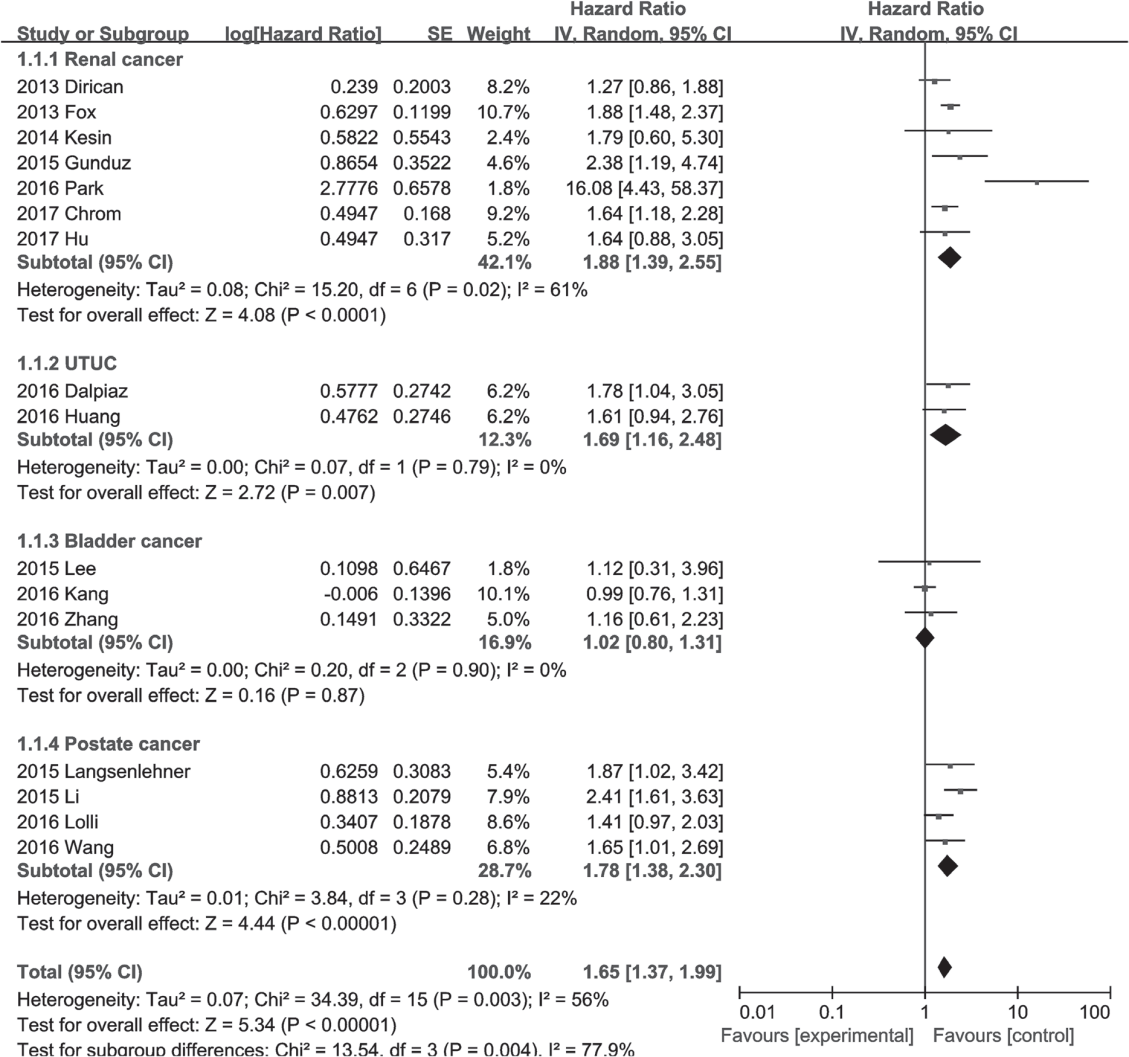
Forest plot of the hazard ratio for the association between an elevated platelet to lymphocyte ratio and overall survival in patients with urologic cancer

**Figure 3:**
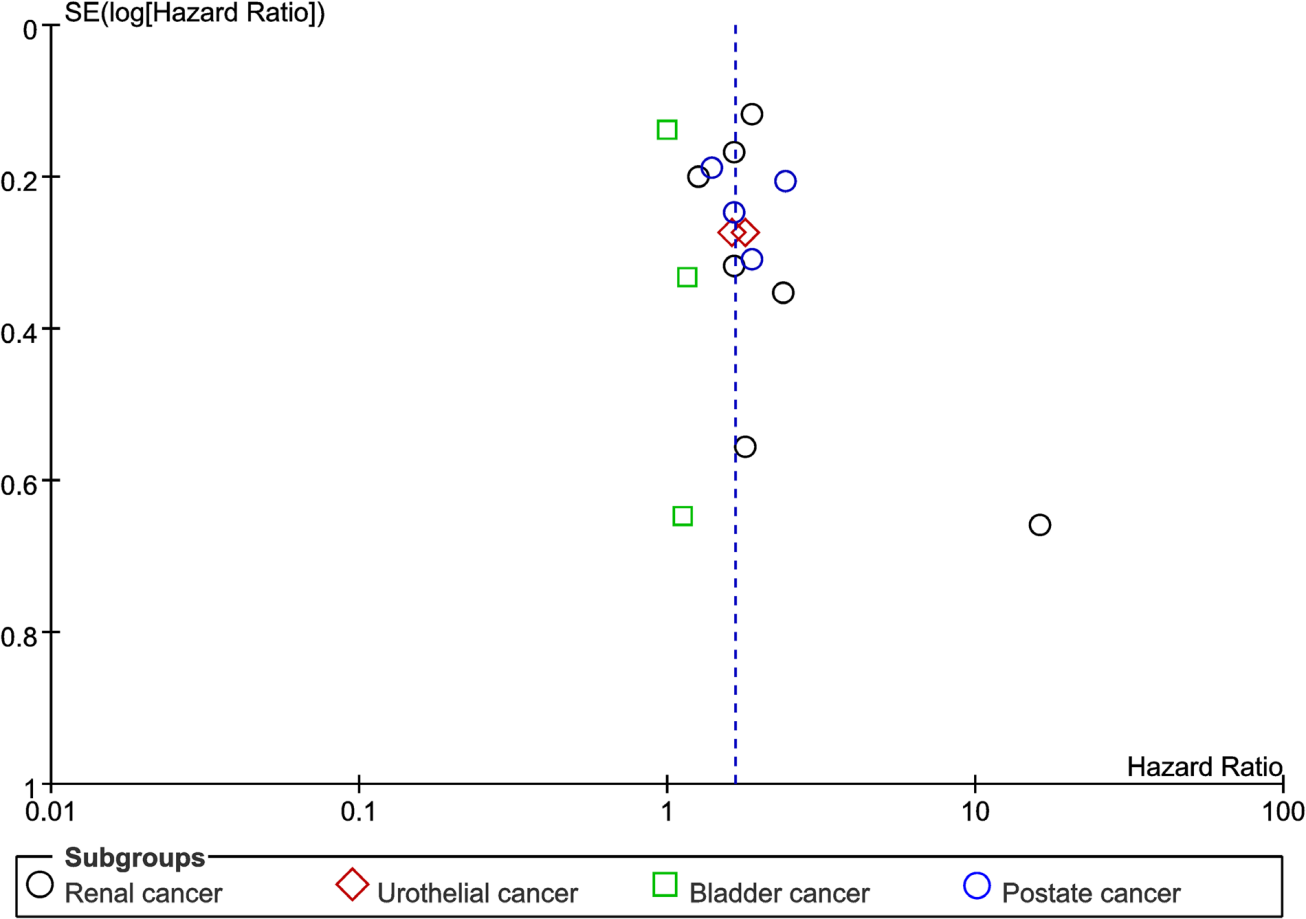
Assessment of publication bias using funnel plot analysis

Sixteen studies evaluated the prognostic role of PLR for OS in urologic cancer. The result indicated that a high PLR was significantly associated with worse prognosis of OS in urologic cancer (HR=1.65, 95% CI=1.37-1.99, P<0.01, I^2^=56%, Figure 2). In addition, the almost symmetrical funnel plot confirmed the absence of publication bias in our study (Figure 3).

### PLR and renal cancer

One study by Lucca et al.[19] reported that an elevated PLR was not significantly associated with poor DFS in patients with renal cancer (HR=1.78, 95% CI=0.87-3.64, P=0.11). A total of seven studies assessed the association between PLR and OS in renal cancer. The result showed that an elevated PLR was significantly associated with poor OS in renal cancer (HR=1.88, 95% CI=1.39-2.55, P<0.01, I^2^=61%, Figure 2). Subgroup analysis revealed that the significant association between poor OS and elevated PLR can be observed in Western population (HR=1.71, 95% CI=1.45-2.02, P<0.01, I^2^=0%), but not in Asian populations (HR=4.77, 95% CI=0.51-44.52, P=0.17, I^2^=90%, Table 2). In other subgroup analysis, the significant association between poor OS and elevated PLR in renal cancer was consistent regardless of treatment, cut-off value, sample size and study quality (Table 2).

**Table 2:** Results of overall and subgroup analyses for effects of PLR on overall survival in renal cancer

Categories	N	Patients	Pooled HR (95% CI)	*P* value	Heterogeneity	
					I^2^ (%)	Ph
Overall effect	7	1594	1.88 (1.39-2.55)	<0.01	61	0.02
Region						
Asian countries	2	547	4.77 (0.51-44.52)	0.17	90	<0.01
Western countries	5	1047	1.71 (1.45-2.02)	<0.01	0	0.44
Treaments						
Non-sugery	3	525	3.31 (1.37-8.01)	<0.01	81	<0.01
Surgery	3	1016	1.65 (1.25-2.18)	<0.01	0	0.99
Cut-off value						
≤157	4	648	2.15 (1.13-4.10)	0.02	78	<0.01
>157	3	946	1.89 (1.53-2.33)	<0.01	0	0.73
Sample size						
≤200	3	216	3.09 (1.01-9.47)	0.048	86	<0.01
>200	4	1378	1.78 (1.49-2.13)	<0.01	0	0.92
Study quality						
≤6	5	748	2.14 (1.27-3.62)	<0.01	73	<0.01
>6	2	846	1.85 (1.48-2.30)	<0.01	0	0.69

95%CI: 95% confidence interval; HR: hazard ratio; N: number of studies; Ph: p value of Q test for heterogeneity test.

On the other hand, two studies presented the data on PLR and PFS in metastatic renal cell cancer. This result is similar to that in OS (HR=4.37, 95% CI=2.58-7.40, P<0.01, I^2^=87%, Supplementary Figure 1).

### PLR and UTUC

Two studies assessed the relationship between PLR and OS, CSS, while another two reported the association between PLR and DFS in patients with UTUC. These results indicated that a high PLR was significantly correlated with poor OS (HR=1.69, 95% CI=1.16-2.48, P<0.01, I^2^=0%, Figure 2) and CSS (HR=1.74, 95% CI=1.11-2.71, P=0.01, I^2^=0%, Supplementary Figure 2), and we found that an elevated PLR tended to be associated with poor DFS (HR=1.46, 95% CI=0.95-2.25, P=0.09, I^2^=0%).

### PLR and bladder cancer

There were three studies reporting the data on PLR and OS in bladder cancer. This result showed that an elevated PLR was not significantly correlated with poor OS (HR=1.02, 95% CI=0.80-1.31, P=0.87, I^2^=0%, Figure 2).

### PLR and prostate cancer

Four studies presented the data on PLR and OS, while two studies reported the data on PLR and CSS in patients with prostate cancer. We found that an elevated PLR predicted shorter OS (HR=1.78, 95% CI=1.38-2.30, P<0.01, I^2^=22%, Figure 2) and CSS (HR=2.02, 95% CI=1.24-3.29, P<0.01, I^2^=31%, Supplementary Figure 2) in prostate cancer.

### PLR and adrenal cancer

There was only one study assessing the association between PLR and prognosis in adrenal cancer. The result of Bagante [34] showed that a high PLR was not significantly associated with poor RFS (HR=1.72, 95% CI=0.96-3.09, P=0.07) and DSS (HR=0.90, 95% CI=0.47-1.73, P=0.76) in adrenal cancer patients.

## DISCUSSION

The prognostic role of PLR has been reported in many types of cancers. However, the prognostic value of PLR in urologic cancer patients is still not unclear. To the best of our knowledge, this is the first pooled study to systematically explore the prognostic significance of PLR in patients with urologic cancer.

In our study, the results indicated that an elevated PLR was significantly associated with poor OS and PFS in renal cancer. Moreover, the significant relationship between poor OS and elevated PLR in renal cancer was consistent regardless of treatment, cut-off value, sample size and study quality. Meanwhile, similar results can be observed in UTUC and prostate cancer. Given all this, PLR is a promising prognostic indicator.

The mechanisms about the association between high PLR and poor prognosis of cancer still remain unknown. Growing evidence has reported that platelets can prevent death of cancer cells by natural killer cells, and can secrete angiogenic and tumor growth factors to promote cancer growth, progression and metastasis [35–37]. Furthermore, it has been reported that thrombocytosis is associated with poor prognosis in renal cancer [38, 39]. While lymphocytes are the main components of immune system in the host and can destroy tumor cells and prevent cancer progression [40]. In addition, some studies reported that a low lymphocyte count was an indicator of poor prognostic in patients with renal cancer [41, 42]. Therefore, a high PLR, which means relatively elevated platelets counts, and low lymphocyte counts may predict poor prognosis in renal cancer.

In this study, most of cohort studies which focus on the role of PLR in renal cancer in this review are from Western countries. Our result indicated that in renal cancer, the significant association between poor OS and elevated PLR can be observed in Western population, but not in Asian populations. It is noted that only two studies performed in Asian countries were included in our study. More future studies should be performed to elucidate the prognostic role of PLR in renal cancer for Asian populations.

On the other hand, in this study, our result showed that an elevated PLR was significantly associated with poor OS in renal cancer and prostate cancer. But the significant association could not been observed in bladder cancer. We think that compared with renal cancer and prostate cancer, bladder cancer is a relatively localized disease. Thus, renal cancer and prostate cancer may be more influenced by the systemic inflammatory response than bladder cancer. Though our result showed a significant association between an elevated PLR and poor OS in UTUC, we should note that only 2 studies about UTUC and 3 studies about bladder cancer were included in our study. More studies are required to confirm the role of PLR in bladder cancer and UTUC in the future.

NLR is a well-known indicator for prognosis in cancer patients. NLR was reported to may represent a balance between procancer inflammatory reaction and anticancer immune function [43]. NLR have been reported to be a prognostic predictor of urologic tumors such as bladder cancer, renal cell cancer, UTUC and prostate cancer [6–10]. While our result indicated that an elevated PLR was not significantly associated with poor OS in bladder cancer. We think that neutrophils may play a more important role in cancer prognosis than platelets, thus may partly explain this result, and it needs to be confirmed in the future.

In our study, the cut-off value PLR in these included studies varied from 117.58 to 241.2. Controversy still exists on the optimal cut-off value of PLR in predicting prognosis for cancer patients. In this present study, we split studies which focus on renal cancer into two groups according to the median value of PLR, the results confirmed that a low PLR is a poor prognostic predictor of OS in both groups. High-quality and well-designed studies are required in the future to set the optimal cut-off value of PLR.

Several limitations exist in our study. First, all these included studies were retrospective studies. Second, heterogeneity among these studies were relatively large and this might be caused by different countries, different types of cancers or/and other factors. Third, due to the related limited number of included studies, we were not able to perform other subgroup analyses.

In conclusion, an elevated PLR was significantly associated with poor survival in renal cancer and prostate cancer. Future studies are warranted to further clarify this association in UTUC, bladder cancer and adrenal cancer.

## MATERIALS AND METHODS

### Search strategy

Two authors (Jianfeng Wang And Jianbin Bi) searched PubMed, Embase, and Web of Science independently for relevant articles published up to March 5, 2017. The main terms were [(bladder OR vesical OR renal OR kidney OR prostate OR prostatic OR urothelial OR urothelium OR adrenal OR urinary OR urology OR urologic) AND (cancer OR neoplasm OR carcinoma OR malignancy)] and (“platelet-lymphocyte ratio” OR “platelet to lymphocyte ratio” OR “platelet lymphocyte ratio” OR PLR). Moreover, potentially searches were also performed by screening the references of relevant review or selected articles.

### Inclusion and exclusion criteria

In this study, PICO criteria (population, intervention, comparison and outcomes) was used to select eligible articles: (1) population: patients who were diagnosed with urologic cancer based on histopathologic examination; (2) intervention: pretreatment or preoperative PLR; (3) comparison: elevated PLR vs. low PLR; (4) outcomes: cancer-specific survival (CSS), disease-free survival (DFS), metastases–free survival (MFS), overall survival (OS), progression-free survival (PFS) and/or recurrence-free survival (RFS). Studies were not included if it was impossible to estimate outcomes from their original data. Case reports and abstracts from meetings were excluded.

### Data extraction

The following data was extracted from each study: name of first author, year of publication, country of patients, sample size, patient characteristics (including gender, age, type of cancer, duration of follow-up and tumor stage), treatment details, cut-off value of PLR and hazard ratio (HR) with associated 95% confidence intervals (CI) for survival.

### Statistical analysis

The pooled HR and 95 % CI were estimated using the inverse variance method with the Random-effects model. The method of Tierney was used to estimate the HR and 95% CI for those studies in which the HR cannot be extracted directly [44]. Cochran’s Q test and I^2^ statistics was used to assess statistical heterogeneity in this study [45]. The Newcastle-Ottawa quality assessment scale (NOS) was used to assess the quality of the studies [46]. Publication bias was evaluated by funnel plot. All the data analyses were conducted using the Review Manager 5.2 software. A P value less than 0.05 was considered as statistically significant.

## SUPPLEMENTARY MATERIALS FIGURES



## Abbreviations

CI: confidence interval
CSS: cancer-specific survival
DFS: disease-free survival
HR: hazard ratio
MFS: metastases–free survival
NLR: neutrophil to lymphocyte ratio
NOS: The Newcastle-Ottawa quality assessment scale
OS: overall survival
PFS: progression-free survival
PLR: platelet to lymphocyte ratio
RFS: recurrence-free survival
UTUC: upper tract urothelial cancer

## References

[1] Siegel RL, Miller KD, Jemal A (2017). Cancer statistics 2017. CA Cancer J Clin.

[2] Williams PA (1987). The role of staging in urologic cancer. Physical assessment and risk factors. Cancer.

[3] Balkwill F, Mantovani A (2001). Inflammation and cancer: back to Virchow?. Lancet.

[4] Mantovani A, Allavena P, Sica A, Balkwill F (2008). Cancer-related inflammation. Nature.

[5] McMillan DC (2009). Systemic inflammation, nutritional status and survival in patients with cancer. Curr Opin Clin Nutr Metab Care.

[6] Luo Y, She DL, Xiong H, Fu SJ, Yang L (2015). Pretreatment neutrophil to lymphocyte ratio as a prognostic predictor of urologic tumors: a systematic review and meta-analysis. Medicine.

[7] Hu K, Lou L, Ye J, Zhang S (2015). Prognostic role of the neutrophil-lymphocyte ratio in renal cell carcinoma: a meta-analysis. BMJ Open.

[8] Wei Y, Jiang YZ, Qian WH (2014). Prognostic role of NLR in urinary cancers: a meta-analysis. PLoS One.

[9] Li X, Ma X, Tang L, Wang B, Chen L, Zhang F, Zhang X (2017). Prognostic value of neutrophil-to-lymphocyte ratio in urothelial carcinoma of the upper urinary tract and bladder: a systematic review and meta-analysis. Oncotarget.

[10] Cao J, Zhu X, Zhao X, Li XF, Xu R (2016). Neutrophil-to-lymphocyte ratio predicts PSA response and prognosis in prostate cancer: a systematic review and meta-analysis. PLoS One.

[11] Ding N, Pang Z, Shen H, Ni Y, Du J, Liu Q (2016). The prognostic value of PLR in lung cancer, a meta-analysis based on results from a large consecutive cohort. Sci Rep.

[12] Guo YH, Sun HF, Zhang YB, Liao ZJ, Zhao L, Cui J, Wu T, Lu JR, Nan KJ, Wang SH (2017). The clinical use of the platelet/lymphocyte ratio and lymphocyte/monocyte ratio as prognostic predictors in colorectal cancer: a meta-analysis. Oncotarget.

[13] Xu Z, Xu W, Cheng H, Shen W, Ying J, Cheng F, Xu W (2016). The prognostic role of the platelet-lymphocytes ratio in gastric cancer: a meta-analysis. PLoS One.

[14] Zhu Y, Si W, Sun Q, Qin B, Zhao W, Yang J (2017). Platelet-lymphocyte ratio acts as an indicator of poor prognosis in patients with breast cancer. Oncotarget.

[15] Dirican A, Kucukzeybek Y, Somali I, Erten C, Demir L, Can A, Bahriye Payzin K, Vedat Bayoglu I, Akyol M, Koseoglu M, Alacacioglu A, Oktay Tarhan M (2013). The association of hematologic parameters on the prognosis of patients with metastatic renal cell carcinoma. J BUON.

[16] Fox P, Hudson M, Brown C, Lord S, Gebski V, De Souza P, Lee CK (2013). Markers of systemic inflammation predict survival in patients with advanced renal cell cancer. Br J Cancer.

[17] Keskin S, Keskin Z, Taskapu HH, Kalkan H, Kaynar M, Poyraz N, Toy H (2014). Prognostic value of preoperative neutrophil-to-lymphocyte and platelet-to-lymphocyte ratios, and multiphasic renal tomography findings in histological subtypes of renal cell carcinoma. BMC Urol.

[18] Gunduz S, Mutlu H, Tural D, Yildiz O, Uysal M, Coskun HS, Bozcuk H (2015). Platelet to lymphocyte ratio as a new prognostic for patients with metastatic renal cell cancer. Asia Pac J Clin Oncol.

[19] Lucca I, de Martino M, Hofbauer SL, Zamani N, Shariat SF, Klatte T (2015). Comparison of the prognostic value of pretreatment measurements of systemic inflammatory response in patients undergoing curative resection of clear cell renal cell carcinoma. World J Urol.

[20] Park TJ, Cho YH, Chung HS, Hwang EC (2016). Prognostic significance of platelet-lymphocyte ratio in patients receiving first-line tyrosine kinase inhibitors for metastatic renal cell cancer. Springerplus.

[21] Chrom P, Stec R, Bodnar L, Szczylik C (2017). Incorporating neutrophil-to-lymphocyte ratio and platelet-to-lymphocyte ratio in place of neutrophil count and platelet count improves prognostic accuracy of the international metastatic renal cell carcinoma database consortium model. Cancer Res Treat.

[22] Hu H, Yao X, Xie X, Wu X, Zheng C, Xia W, Ma S (2017). Prognostic value of preoperative NLR, dNLR, PLR and CRP in surgical renal cell carcinoma patients. World J Urol.

[23] Kim M, Moon KC, Choi WS, Jeong CW, Kwak C, Kim HH, Ku JH (2015). Prognostic value of systemic inflammatory responses in patients with upper urinary tract urothelial carcinoma. World J Urol.

[24] Huang J, Yuan Y, Wang Y, Zhang J, Kong W, Chen H, Chen Y, Huang Y (2017). Prognostic value of preoperative plasma fibrinogen level and platelet-to-lymphocyte ratio (F-PLR) in patients with localized upper tract urothelial carcinoma. Oncotarget.

[25] Dalpiaz O, Krieger D, Ehrlich GC, Pohlmann K, Stojakovic T, Pummer K, Zigeuner R, Pichler M, Hutterer GC (2017). Validation of the preoperative platelet-to-lymphocyte ratio as a prognostic factor in a European cohort of patients with upper tract urothelial carcinoma. Urol Int.

[26] Song X, Zhang GM, Ma XC, Luo L, Li B, Chai DY, Sun LJ (2016). Comparison of preoperative neutrophil-lymphocyte, lymphocyte-monocyte, and platelet-lymphocyte ratios in patients with upper urinary tract urothelial carcinoma undergoing radical nephroureterectomy. Onco Targets Ther.

[27] Lee SM, Russell A, Hellawell G (2015). Predictive value of pretreatment inflammation-based prognostic scores (neutrophil-to-lymphocyte ratio, platelet-to-lymphocyte ratio, and lymphocyte-to-monocyte ratio) for invasive bladder carcinoma. Korean J Urol.

[28] Zhang GM, Zhu Y, Luo L, Wan FN, Zhu YP, Sun LJ, Ye DW (2015). Preoperative lymphocyte-monocyte and platelet-lymphocyte ratios as predictors of overall survival in patients with bladder cancer undergoing radical cystectomy. Tumour Biol.

[29] Kang M, Jeong CW, Kwak C, Kim HH, Ku JH (2017). Preoperative neutrophil-lymphocyte ratio can significantly predict mortality outcomes in patients with non-muscle invasive bladder cancer undergoing transurethral resection of bladder tumor. Oncotarget.

[30] Langsenlehner T, Pichler M, Thurner EM, Krenn-Pilko S, Stojakovic T, Gerger A, Langsenlehner U (2015). Evaluation of the platelet-to-lymphocyte ratio as a prognostic indicator in a European cohort of patients with prostate cancer treated with radiotherapy. Urol Oncol.

[31] Li F, Hu H, Gu S, Chen X, Sun Q (2015). Platelet to lymphocyte ratio plays an important role in prostate cancer's diagnosis and prognosis. Int J Clin Exp Med.

[32] Lolli C, Caffo O, Scarpi E, Aieta M, Conteduca V, Maines F, Bianchi E, Massari F, Veccia A, Chiuri VE, Facchini G, De Giorgi U (2016). Systemic immune-inflammation index predicts the clinical outcome in patients with mCRPC treated with abiraterone. Front Pharmacol.

[33] Wang Y, Xu F, Pan J, Zhu Y, Shao X, Sha J, Wang Z, Cai Y, Liu Q, Dong B, Xue W, Huang Y (2016). Platelet to lymphocyte ratio as an independent prognostic indicator for prostate cancer patients receiving androgen deprivation therapy. BMC Cancer.

[34] Bagante F, Tran TB, Postlewait LM, Maithel SK, Wang TS, Evans DB, Hatzaras I, Shenoy R, Phay JE, Keplinger K, Fields RC, Jin LX, Weber SM (2015). Neutrophil-lymphocyte and platelet-lymphocyte ratio as predictors of disease specific survival after resection of adrenocortical carcinoma. J Surg Oncol.

[35] Palumbo JS, Talmage KE, Massari JV, La Jeunesse CM, Flick MJ, Kombrinck KW, Jirouskova M, Degen JL (2005). Platelets and fibrin (ogen) increase metastatic potential by impeding natural killer cell-mediated elimination of tumor cells. Blood.

[36] Gay LJ, Felding-Habermann B (2011). Contribution of platelets to tumour metastasis. Nat Rev Cancer.

[37] Suzuki K, Aiura K, Ueda M, Kitajima M (2004). The influence of platelets on the promotion of invasion by tumor cells and inhibition by antiplatelet agents. Pancreas.

[38] Choi JY, Ko YH, Song PH (2016). Clinical significance of preoperative thrombocytosis in patients who underwent radical nephrectomy for nonmetastatic renal cell carcinoma. Investig Clin Urol.

[39] Men H, Liang C, Yu M (2015). Thrombocytosis as a prognostic factor in patients with renal cell carcinoma: a meta-analysis of literature. J Cancer Res Ther.

[40] Rosenberg SA (2001). Progress in human tumour immunology and immunotherapy. Nature.

[41] De Giorgi U, Rihawi K, Aieta M, Lo Re G, Sava T, Masini C, Baldazzi V, De Vincenzo F, Camerini A, Fornarini G, Burattini L, Rosti G, Moscetti L (2014). Lymphopenia and clinical outcome of elderly patients treated with sunitinib for metastatic renal cell cancer. J Geriatr Oncol.

[42] Mehrazin R, Uzzo RG, Kutikov A, Ruth K, Tomaszewski JJ, Dulaimi E, Ginzburg S, Abbosh PH, Ito T, Corcoran AT, Chen DY, Smaldone MC, Al-Saleem T (2015). Lymphopenia is an independent predictor of inferior outcome in papillary renal cell carcinoma. Urol Oncol.

[43] Li MX, Liu XM, Zhang XF, Zhang JF, Wang WL, Zhu Y, Dong J, Cheng JW, Liu ZW, Ma L, LV Y (2014). Prognostic role of neutrophil-to-lymphocyte ratio in colorectal cancer: a systematic review and meta-analysis. Int J Cancer.

[44] Tierney JF, Stewart LA, Ghersi D, Burdett S, Sydes MR (2007). Practical methods for incorporating summary time-to-event data into meta-analysis. Trials.

[45] Higgins JP, Thompson SG, Deeks JJ, Altman DG (2003). Measuring inconsistency in meta-analyses. BMJ.

[46] Stang A (2010). Critical evaluation of the Newcastle-Ottawa scale for the assessment of the quality of nonrandomized studies in meta-analyses. Eur J Epidemiol.

