# Supplementary material for: Prognostic role of pretreatment platelet to lymphocyte ratio in urologic cancer
Source: Oncotarget. 2017 Aug 10;8(41):70874–82. doi: 10.18632/oncotarget.20147 (PMC5642603; doi:10.18632/oncotarget.20147)
Supplement: Supplementary file 1 [file oncotarget-08-70874-s001.pdf]

Prognostic role of pretreatment platelet to lymphocyte ratio in urologic cancer

SUPPLEMENTARY MATERIALS

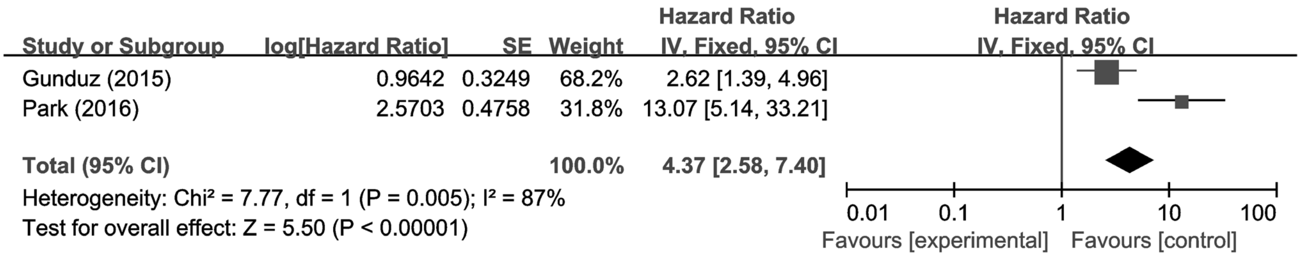

Supplementary Figure 1: Forest plot of the hazard ratio for the association between an elevated platelet to lymphocyte ratio and progression-free survival in renal cancer.

Study or Subgroup	log[Hazard Ratio]	SE	Weight	Hazard Ratio IV, Fixed, 95% CI
**1.2.1 UTUC**				
2016 Dalpiaz	0.4253	0.3059	54.9%	1.53 [0.84, 2.79]
2016 Huang	0.7061	0.3378	45.1%	2.03 [1.05, 3.93]
**Subtotal (95% CI)**			**100.0%**	**1.74 [1.11, 2.71]**
Heterogeneity: Chi² = 0.38, df = 1 (P = 0.54); I² = 0%				
Test for overall effect: Z = 2.43 (P = 0.01)				
**1.2.2 Postate cancer**				
2015 Langsenlehner	1.3838	0.6173	16.3%	3.99 [1.19, 13.38]
2016 Wang	0.5697	0.2725	83.7%	1.77 [1.04, 3.02]
**Subtotal (95% CI)**			**100.0%**	**2.02 [1.24, 3.29]**
Heterogeneity: Chi² = 1.46, df = 1 (P = 0.23); I² = 31%				
Test for overall effect: Z = 2.82 (P = 0.005)				

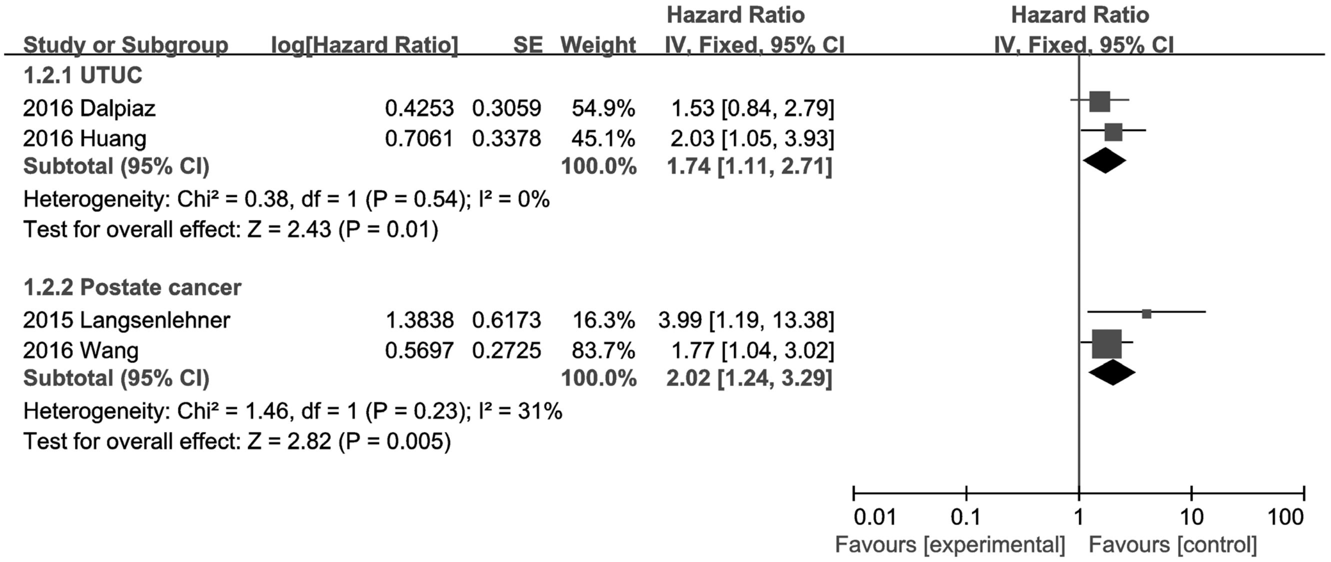

Supplementary Figure 2: Forest plot of the hazard ratio for the association between an elevated platelet to lymphocyte ratio and cancer-specific survival in UTUC and prostate cancer.
